# Tubular endocytosis drives remodelling of the apical surface during epithelial morphogenesis in *Drosophila*

**DOI:** 10.1038/ncomms3244

**Published:** 2013-08-07

**Authors:** Piotr Fabrowski, Aleksandar S. Necakov, Simone Mumbauer, Eva Loeser, Alessandra Reversi, Sebastian Streichan, John A. G. Briggs, Stefano De Renzis

**Affiliations:** 1 Department of Developmental Biology, European Molecular Biology Laboratory (EMBL) Heidelberg, Meyerhofstrasse 1, Heidelberg 69117, Germany; 2 Department of Structural and Computational Biology, European Molecular Biology Laboratory (EMBL) Heidelberg, Meyerhofstrasse 1, Heidelberg 69117, Germany; 3 These authors contributed equally to this work

## Abstract

During morphogenesis, remodelling of cell shape requires the expansion or contraction of plasma membrane domains. Here we identify a mechanism underlying the restructuring of the apical surface during epithelial morphogenesis in *Drosophila*. We show that the retraction of villous protrusions and subsequent apical plasma membrane flattening is an endocytosis-driven morphogenetic process. Quantitation of endogenously tagged GFP::Rab5 dynamics reveals a massive increase in apical endocytosis that correlates with changes in apical morphology. This increase is accompanied by the formation of tubular plasma membrane invaginations that serve as platforms for the *de novo* generation of Rab5-positive endosomes. We identify the Rab5-effector Rabankyrin-5 as a regulator of this pathway and demonstrate that blocking dynamin activity results in the complete inhibition of tubular endocytosis, in the disappearance of Rab5 endosomes, and in the inhibition of surface flattening. These data collectively demonstrate a requirement for endocytosis in morphogenetic remodelling during epithelial development.

## Abstract (web summary)

During epithelial morphogenesis in Drosophila, the villous apical cell surface is flattened. Fabrowski *et al*. show that this flattening depends on a dramatic increase in endocytosis associated with the formation of tubular invaginations, revealing a role for membrane trafficking in morphological remodelling.

## Graphical abstract

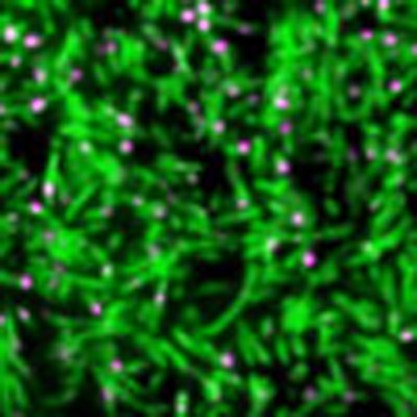

Remodelling of epithelial tissues is often driven by changes in apical surface area and membrane tension in single cells or in groups of cells. Flattening and constriction of the apical surface drives tissue invagination, while plasma membrane stretching and elongation is commonly observed during tissue elongation^1^,^2^,^3^,^4^. During epithelial differentiation, the apical surface also undergoes more complex structural and functional modes of reorganization that bring about specialized functions such as absorption, secretion and photo-transduction^5^,^6^. A large body of experimental evidence has demonstrated the importance of the cytoskleton in regulating the structure and dynamics of the plasma membrane. However, the high level of membrane turnover during morphological remodelling suggests that membrane trafficking and in particular transport along the apical endocytic pathway might also have an important role during the biogenesis and re-organization of the apical surface. For example, the internalization and recycling of both adhesion molecules and ion channels regulate tube morphogenesis during airway development^7^.

Apical endocytosis is an heterogeneous process involving different types of tubulo-vesicular intermediates^8^,^9^. Although the molecular mechanisms underlying the biogenesis of different endocytic intermediates are beginning to emerge, their specific function in the context of living organisms and developing epithelia are still poorly understood.

Cellularization of the early *Drosophila* embryo is an ideal *in vivo* system for studying the endocytic mechanisms driving plasma membrane remodelling during epithelial morphogenesis. Cellularization is a specialized form of cytokinesis coupled to the formation of a polarized epithelium^10^,^11^,^12^. Over the course of an hour, a syncitium of 6,000 nuclei is sub-divided into an equal number of mononucleated cells through the invagination and growth of the apical plasma membrane^13^. Endocytosis is required to complete cellularization and is particularly prominent at invaginating furrows^14^. However, it has also been suggested that apical endocytosis supports furrow ingression by controlling the redistribution of membranes from the apical to the basolateral surface^15^. In agreement with this proposal, blocking the early endocytic regulators Rab5 and Rab11 results in the arrest of furrow ingression. A similar block in cellularization was also observed in *shibire*^*ts*^ mutant embryos at the non-restrictive temperature (*shibire*^*ts*^ is a temperature-sensitive allele of dynamin, a large GTPase that controls scission of clathrin-coated vesicles from the plasma membrane^15^).

During cellularization, the apical plasma membrane undergoes a dramatic morphological transformation. At the beginning of cellularization, the apical surface is rich in highly dynamic membrane protrusions that undergo a progressive, stereotypical retraction and flattening as cellularization proceeds^16^. This change in apical morphology is concomitant with a corresponding change in the organization of the underlying actin cytoskeleton and the establishment of adherens junctions that serve to interconnect the cellular epithelium. Mutations in the proto-oncogenic kinase Abelson, a key regulator of adherens junction stability and actin organization, result in an excess of actin polymerization in membrane protrusions and a corresponding delay in surface flattening^17^.

These stereotypic changes in apical morphology provide a unique opportunity to study the spatio-temporal organization of apical endocytosis and its relationship to different plasma membrane states during epithelial morphogenesis. To date, however, the apical surface of the embryo has proven extremely difficult to visualize in real time using epifluorescence or confocal live imaging techniques. Here we have employed a modified form of total internal reflection fluorescence (TIRF) microscopy in live embryos to visualize distinct components of the endocytic machinery and their interaction with the plasma membrane during surface flattening with unprecedented resolution. We used homologous recombination to tag the early endocytic regulator Rab5 with enhanced green fluorescent protein (EGFP) at its endogenous locus and followed the dynamics of apical endosomes under physiological expression levels. Rab5 is a small GTPase of the Ras superfamily that is localized to the plasma membrane, clathrin-coated vesicles and early endosomes^18^. In polarized epithelial cells, Rab5 localizes to both basal and apical endosomes^19^ and, in its GTP-bound conformation, interacts with over 20 different proteins, termed Rab5 effectors. These effectors form distinct macromolecular complexes on apical and basal endosomes^20^,^21^. Importantly, Rab5 is rate-limiting for both basal and apical endocytosis^19^. Thus, Rab5 and its effectors are excellent molecular tools for following the machinery underlying endosome biogenesis and function during cell and tissue morphogenesis.

In summary, our data show that the activation of a dynamin-dependent apical endocytic pathway involving the formation of long tubular intermediates, labelled by Rab5 and its effector Rabankyrin-5, controls the remodelling of the apical plasma membrane during cellularization. Collectively, these results provide a direct link between a specific apical endocytic pathway and remodelling of plasma membrane structure during epithelial morphogenesis, and suggest the presence of a general regulatory mechanism linking changes in plasma membrane morphology and endocytosis during development.

## Results

### TIRF imaging of apical membrane dynamics in live embryos

**Figure 1:**
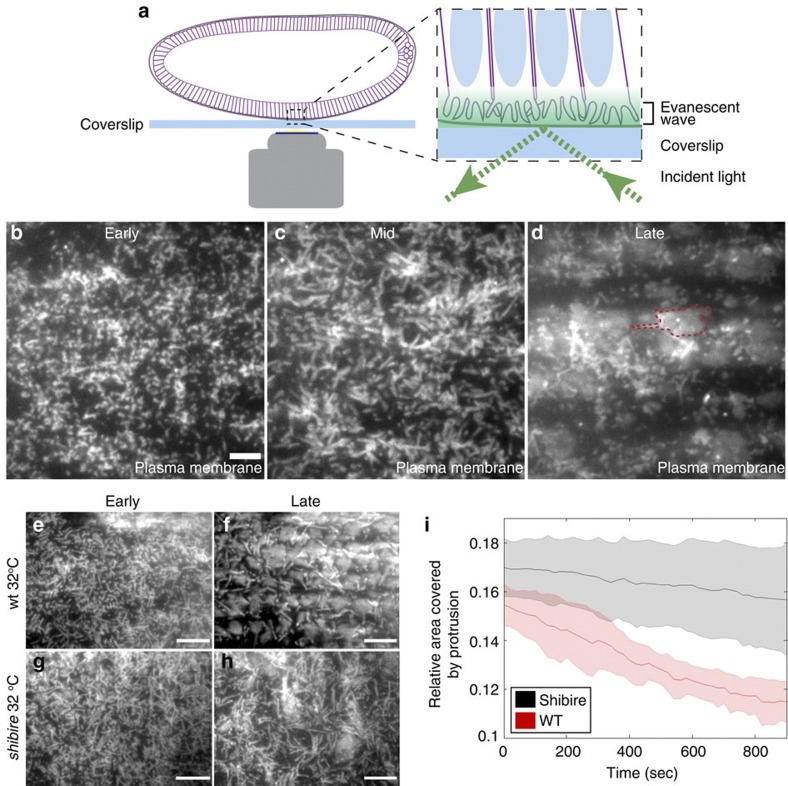
Endocytosis drives plasma membrane flattening during epithelial morphogenesis. (**a**) Schematic overview of TIRF imaging in the early *Drosophila* embryo. Embryos (cell membranes outlined in purple) are mounted such that the evanescent wave (green gradient) generated at the coverslip (blue rectangle) by total internal reflection of incident excitation light (dotted green lines) illuminates the apical plasma membrane (shown as purple protrusions). Nuclei are depicted as blue ovals. (**b**–**d**) Apical view of cellularizing embryos expressing GAP43::mCherry using TIRF microscopy at progressive stages through cellularization (**b**=early (~5 min. into cellularization); **c**=middle (~20 min. into cellularization); **d**=late (~40 min. into cellularization). During the early phase of cellularization, the apical plasma membrane is covered with small fillopodial-like protrusions (**b**) that thicken and elongate as cellularization proceeds. These protrusions progressively retract as the apical plasma membrane flattens (dotted red outline) towards the end of cellularization (**d**). Scale bar, 5 μm. (**e**–**h**) TIRF-M imaging of the apical plasma membrane in wt (**e**,**f**) and *shibire* mutant (**g**,**h**) embryos reared at 32 °C over the course of cellularization. Progressive flattening of the apical plasma membrane was observed in wild-type embryos (**f**), whereas surface flattening was impeded in *shibire* mutant embryos (**h**). Scale bar, 10 μm. (**i**) Quantification of the fractional surface area coverage by protrusions in wild-type (pink line, ±s.d.) and *shibire* mutant (blue line, ±s.d.) embryos. Time zero corresponds to the time point at which embryos at mid-cellularization were shifted to the restrictive temperature (32 °C). The *P*-value calculated using a KS-test (*n*=3 for both WT and *shibire*) between conditions at the end of acquisition=1.237 × 10^−31^.

Our understanding of the mechanisms underlying the morphogenesis of the apical surface has been limited by the lack of suitable imaging methods to visualize apical membrane dynamics during epithelial development. To circumvent this limitation, we applied TIRF microscopy (TIRF-M) to cellularizing *Drosophila* embryos (Fig. 1a) (Methods). TIRF-M is exceptionally well suited for following membrane dynamics with high spatio-temporal resolution, as it relies upon an evanescent wave that exclusively illuminates a region in close proximity (10–200 nm) to the coverslip^22^.

As a first step, we imaged embryos expressing a fusion protein of the plasma membrane marker peptide GAP43 (amino acids 1–20) tagged with the mCherry fluorescent protein^11^. In accordance with previous electron microscopy data^16^, TIRF-M allowed us to observe a massive accumulation of membranes at the apical surface before cellularization. During the early phase of cellularization, the apical surface of all blastoderm cells was covered with small, highly dynamic, villous protrusions (Fig. 1b and Supplementary Movie 1). Over the course of cellularization, these protrusions first begin to elongate and broaden at mid-cellularization (Fig. 1c), and are ultimately reabsorbed towards the end of cellularization as the apical plasma membrane flattens (Fig. 1d). To exclude the possibility that we were imaging intracellular plasma membrane invaginations rather than apical protrusions, we imaged embryos co-expressing GAP43 and the plus- end microtubule binding protein EB1. As shown in Supplementary Movie 2, EB1 could be clearly seen trafficking inside of GAP43 labelled plasma membrane protrusions. Furthermore, at the super critical angle required for the generation of the evanescent wave only a thin (~0.4 μm) plane was in focus (Supplementary Fig. S1), thus arguing that a *bona fide* TIRF signal was indeed obtained. To further confirm that only the apical plasma membrane was illuminated under this condition, we imaged embryos expressing GAP43 at the beginning of cycle 14, when the domains of the plasma membrane corresponding to the furrows are still wide and have invaginated for only 5 μm. Although apical protrusions were clearly in focus, the area of the plasma membrane corresponding to the furrows was not illuminated, appearing dark and indistinguishable from the extracellular space (Supplementary Fig. S2). We therefore conclude that TIRF-M serves as an excellent approach with which to follow apical plasma membrane dynamics in the cellularizing *Drosophila* embryo.

### Dynamin activity is required for apical flattening

Considering the reduction in apical surface and changes in membrane organization that we observed, we next asked whether membrane flattening is causally linked to endocytosis. One key regulator of endocytosis is the large GTPase dynamin^23^. Dynamin controls the scission of clathrin-coated vesicles from the plasma membrane^24^ and has also been implicated in the biogenesis of a distinct set of endocytic structures, including macropinosomes^25^. Using TIRF-M, we followed the dynamics of the GAP43-mCherry marked apical plasma membrane over the course of cellularization in embryos containing the *shibire*^*ts*^ mutation, a temperature-sensitive allele of dynamin^26^. Whereas wild-type embryos imaged at the restrictive temperature (32 °C) undergo apical surface flattening without any morphological abnormalities (Fig. 1e,f), this process was severely compromised in *shibire*^*ts*^ mutant embryos (Fig. 1g,h). Upon shifting embryos to the restrictive temperature, the apical plasma membrane failed to flatten and the retraction of membrane protrusions did not occur (Fig. 1h,i and Supplementary Movie 3). This result demonstrates that apical surface flattening is dynamin-dependent, suggesting that endocytosis might actively participate in this process.

### Tubular endocytosis is activated during surface flattening

**Figure 2:**
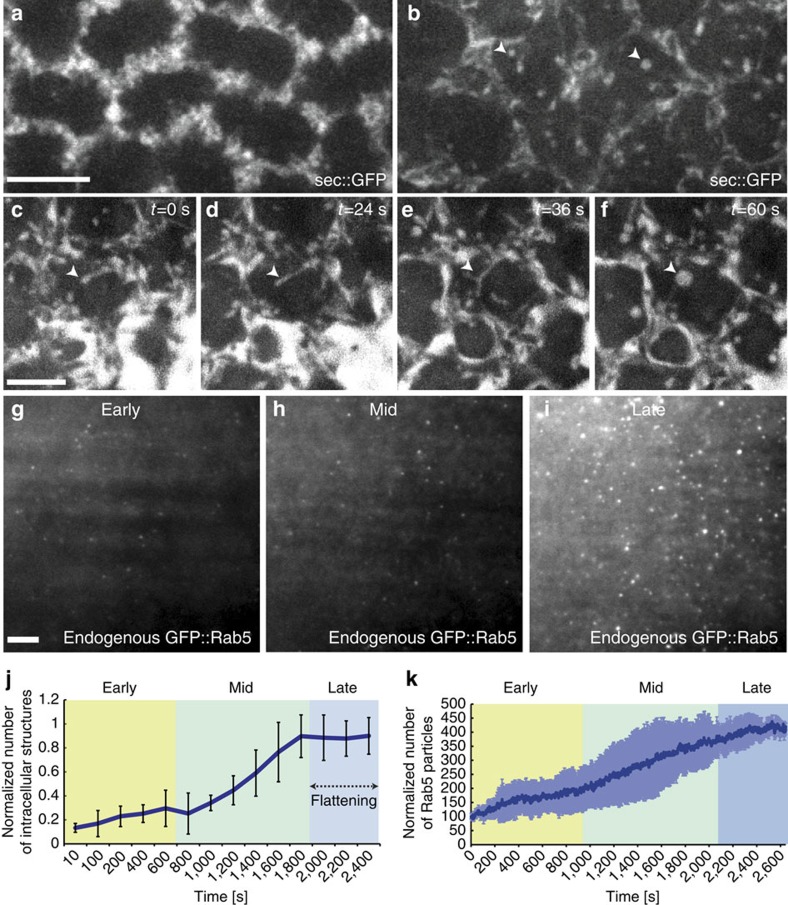
Apical Rab5 endocytosis is upregulated during cellularization and coincides precisely with the timing of apical flattening. (**a**,**b**) Still frames from a time-lapse recording showing the upregulation of sec::GFP internalization. During early cellularization (**a**) sec::GFP localizes primarily to the extracellar space, whereas during late cellularization numerous internal sec:GFP-positive structures could easily be detected (**b**, white arrows) Scale bar, 5 μm. (**c**–**f**) Still frames from a 3.5 μm *z* projection time-lapse recording under the apical surface showing formation of an intracellular vacuole filled with sec::GFP from a tube originating at the plasma membrane. Timepoints correspond to (**c**) *t*=0 s, (**d**) *t*=24 s, (**e**) *t*=36 s, (**f**) t=60 s. (**g**–**i**) Still frames from a time-lapse TIRF recording showing the upregulation of apical endosomal structures marked by endogenously tagged GFP::Rab5. A progressive increase in the number of apical Rab5 puncta between early (**g**), middle (**h**) and late (**i**) cellularization was observed. Scale bar, 5 μm. (**j**) Quantification of sec::GFP-positive endocytic structures (tubules and vacuoles) over the course of cellularization (Blue line) including s.d. (black bars). The early (yellow), mid- (green) and late (purple) stages of cellularization are highlighted. The number of internal sec::GFP-positive structures (tubules and vacuoles) was quantified in five independent *z* sections separated by 0.2 μm, in nine separate 625 μm^2^ surface regions, from three embryos. *P*-value=1.0 × 10^−6^ (ANOVA). (**k**) Quantification of endogenously tagged Rab5 endosomes at the apical surface over the course of cellularization. The early (yellow), mid- (green), and late (purple) stages of cellularization are highlighted. GFP::Rab5 signal intensity is represented as the normalized number of Rab5 particles versus time (dark blue line) including s.d. (light blue bars). *n*=3 embryos. *P*-value=2.2 × 10^−16^ (*t*-test).

To ask whether an increase in the rate of apical endocytosis might account for the decrease in the surface area of the plasma membrane during apical flattening, we developed a quantitative assay for monitoring the uptake of a soluble fluorescent tracer. To this end, we generated a genetically encoded endocytic cargo consisting of EGFP fused to the secretion signal peptide of folded gastrulation (fog), a secreted signalling molecule expressed in early embryos^27^. This fusion protein, henceforth referred to as sec::GFP, is secreted, filling the extracellular space/perivitelline fluid with a soluble, fluorescent endocytic tracer before cellularization. Using this assay, we show that the number of endocytic structures increased approximately fivefold over the course of cellularization, reaching a maximum during surface flattening (Fig. 2a,b,j and Supplementary Movie 4). Strikingly, this experiment also revealed that the only visible entry route for soluble cargo is through tubular intermediates that, upon budding from the plasma membrane, form vacuolar structures (Fig. 2c–f). These structures formed towards the end of cellularization were particularly prominent in ventral cells, and originated from both the apical and the subapical region of the cell.

### Rab5 endosomes are upregulated during surface flattening

**Figure 3:**
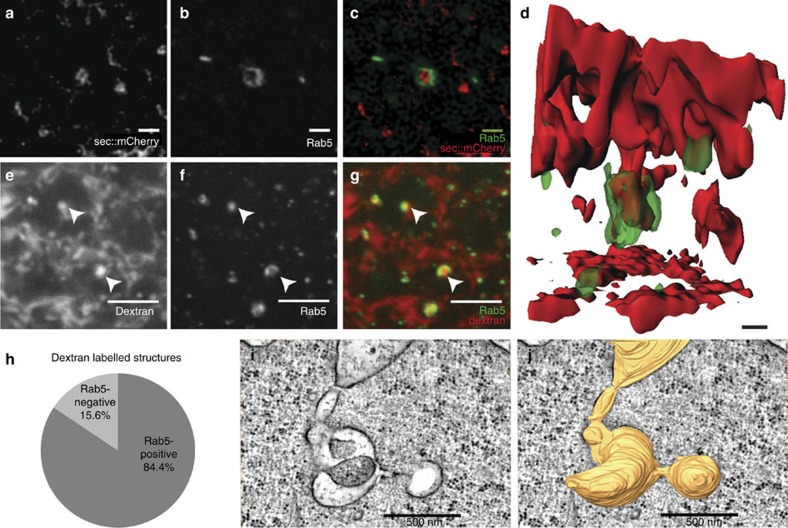
The primary entry route for apical endocytic cargo is through endocytic tubules that interact directly with Rab5. (**a**–**d**) sec::mCherry-positive endocytic structures (**a**) colocalize with endogenously tagged GFP::Rab5 (**b**). GFP::Rab5 (green), sec::mCherry (red) overlay (**c**). (**d**) Three dimensional rendering of a typical apical endocytic tubule labelled by sec::mCherry (red), co-labelled by endogenous GFP::Rab5 (green). Scale bar, 500 nm. (**e**–**h**) Quantification of dextran-labelled subapical structures positive for Rab5. (**e**–**g**) Single-plane confocal images showing subapical intracellular Dextran-positive endocytic structures (**e**, white arrows) that colocalize with endogenously tagged GFP::Rab5 (**f**, white arrows). GFP::Rab5 (green), Dextran (red) overlay (**g**). Scale bar, 5 μm. (**h**) Quantification of the percentage of intracellular dextran-labelled structures positive for Rab5. A total of 100 Dextran-labelled structures were marked in the red channel. The percentage of these structures that colocalized with Rab5 was estimated by counting the number of these structures that were also positive for Rab5 in the green channel (*n*=3 embryos), *P*-value=0.015 (*t*-test). (**i**,**j**) EM tomography of an apical endocytic tubule showing multiple internal compartments in direct continuity with the apical plasma membrane. A single section through an EM tomogram of a tubular-endocytic structure in direct continuity with the apical plasma membrane (**i**). Three-dimensional surface rendering of the tubular-endocytic volume from the corresponding volume generated by EM tomography demonstrating the continuity of the tubular-endosomal structure with the apical plasma membrane at top (**j**). Scale bar, 500 nm.

Given these changes in endocytic activity, we next asked whether the endosomal machinery also undergoes dynamic regulation during apical surface flattening. To this end, we used a homologous recombination-based strategy^28^ to ‘knock in’ an EGFP tag into the endogenous genomic locus of Rab5. TIRF-M imaging revealed that, during early cellularization, relatively few Rab5-positive endosomes exist at the apical plasma membrane (Fig. 2g,h and Supplementary Movie 4). In contrast, and consistent with a role of endocytosis in apical flattening, the number of these Rab5-positive endosomes increased threefold during apical flattening (Fig. 2i,k and Supplementary Movie 5). To verify this result with a different imaging approach, we quantified the number of Rab5 apical endosomes in sagittal cross-section using two-photon microscopy. Using this approach, we measured an approximately fourfold upregulation in the number of Rab5 apical endosomes over the course of cellularization (Supplementary Movie 6 and Supplementary Fig. S3a,b). Moreover, we observed no significant increase in total Rab5 signal intensity over the course of cellularization (Supplementary Fig. S3c,d) thus excluding the possibility that the apical increase in Rab5 resulted simply from an overall increase in expression levels. Simultaneous imaging of GFP::Rab5 and secreted mCherry in sagittal cross-sections (sec::mCherry; a variant of sec::GFP in which the GFP was substituted with mCherry) revealed that Rab5 associates directly with cargo-filled tubular invaginations (Fig. 3a–d). To quantify the extent of colocalization between internalized cargo and Rab5, we injected fluorescent dextran into the perivitelline space of late cellularizing embryos during surface flattening (the signal of internalized sec::mCherry bleaches very quickly thus precluding its use for quantitative analysis). Using this approach, we found that ~80% of dextran-positive structures were co-labelled also by GFP::Rab5 (Fig. 3e–h). The localization of Rab5 to plasma membrane invaginations argues that during surface flattening, apical endosomes originate from these structures and that the Rab5 puncta observed in TIRF (Fig. 2g–i) correspond most likely to the association of Rab5 with the newly formed apical endosomes. We further show using electron tomography that tubular invaginations of the plasma membrane exist as convoluted structures surrounded by an area of ribosomal exclusion, which is suggestive of actin polymerization (Fig. 3i,j).

**Figure 4:**
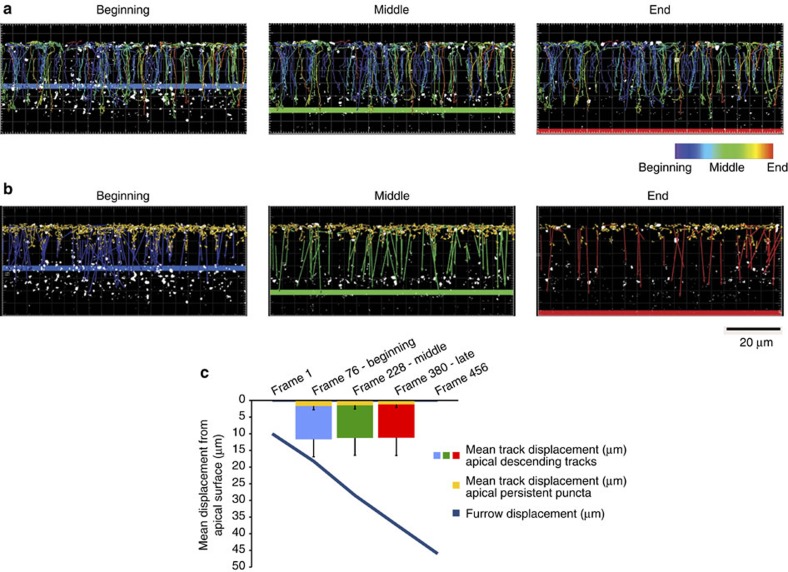
Subcellular Tracking of apical Rab5-positive endosomes reveals prominent apical biogenesis and subsequent basal descent. (**a**,**b**) Tracking analysis was performed on spinning disc confocal time-lapse series of YFP::Rab5-positive endosomes during the late phase of cellularization. All Rab5-positive structures, shown as segmented puncta in white, with an origin within 5 μm of the apical surface, and that could be detected for a minimum duration of six frames were tracked in space over time. (**a**) All identified tracks are shown, coded in colour by time, with the position of the furrow shown at the beginning (blue bar), middle (green bar) and end (red bar) of late/fast phase cellularization. (**b**) The extent of displacement from the apical surface for all tracks during the beginning, middle and end of cellularization. Coloured arrows represent the displacement of individual tracks, and point in the direction of travel over time. Blue, green and red arrows represent tracks that occur during the beginning, middle or end of late/fast phase cellularization, respectively. Yellow tracks represent Rab5-positive endosomes that persist at the apical surface. Furrow positions during the beginning, middle and end of late/fast phase cellularization are shown as blue, green and red bars, respectively. Scale bar, 20 μm. (**c**) Quantification of the length of apical to basal travel with respect to furrow position. The mean track displacement, in microns, for apically formed, basally descending Rab5 endosomes is shown during the beginning (blue), middle (green) and end (red) of the late/fast phase of cellularization. The mean track displacement, in microns, for apically persistent Rab5 endosomes is shown in yellow. Error bars represent the standard deviation of the mean track displacement. The blue line represents furrow position, in microns, with respect to the apical surface over time. *n*=17 cells.

Live imaging further demonstrated that upon leaving from the apical cytoplasm, Rab5 apical endosomes travel basally for ~15 μm, reaching the base of the nuclei in close proximity to the invaginating furrow (Fig. 4a,c and Supplementary Movie 7). However, as cellularization proceeds, the maximal displacement length of individual Rab5 endosomes did not increase, and, in fact, these endosomes form a stable population below the base of the nuclei (Fig. 4c). Thus, the production of Rab5 endosomes does not seem to be directly linked with furrow ingression but rather correlates with flattening of the apical surface.

### Rabankyrin-5 controls tubular-endocytic membrane processing

**Figure 5:**
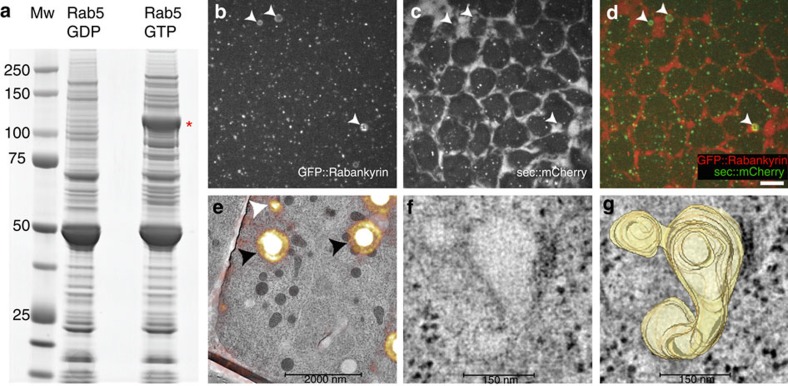
The biogenesis of apical endocytic tubules is regulated by dynamin and the Rab5-effector Rabankyrin-5. (**a**) Coomasie staining of GST-Rab5 affinity chromatography products using 0–4 h embryo cytosolic fractions. Lane 1 (Mw) corresponds to a protein molecular weight ladder (corresponding protein sizes, in kDa, are shown at left). Lanes 2 and 3 (Rab5 GDP, Rab5 GTP) correspond to the proteins bound to GDP-loaded Rab5, and GTP-γ-S-loaded Rab5, respectively. The red asterisk marks the protein band identified by mass spectrometry as CG41099, the *Drosophila* homologue of mammalian Rabankyrin-5. (**b**–**d**) Rabankyrin-5 localizes to Apical Endocytic Structures. Ectopic expression of GFP::Rabankyrin-5 with sec::mCherry shows colocalization of both proteins to apical vacuolar structures (white arrows). Single-plane images of GFP::Rabankyrin-5 (**b**) and sec::mCherry (**c**). Overlay of GFP::Rab5 (green) and sec::mCherry (red) showing extensive colocalization (**d**). (**e**–**g**) Ultrastructural analysis of GFP::Rabankyrin-5-positive structures by correlative light-electron microscopy. Correlative light-electron micrograph showing the overlay of GFP::Rabankyrin-5 fluorescence onto a corresponding EM tomogram of the same region (**e**). A GFP::Rabankyrin-5-positive structure (white arrow) is shown in relation to the fluorescence of TetraSpeck-based fiducials (black arrows). Scale bar, 2,000 nm. (**f**) High-magnification view of a section of the EM tomogram corresponding to the region underlying the GFP::Rabankyrin-5-positive spot labelled in panel **e** (white arrow). (**g**) Three-dimensional surface rendering of the convoluted, tubular-membranous, GFP::Rabankyrin-5-positive structure underlying the fluorescence spot marked in panel a (white arrow). Scale bar, 150 nm.

**Figure 6:**
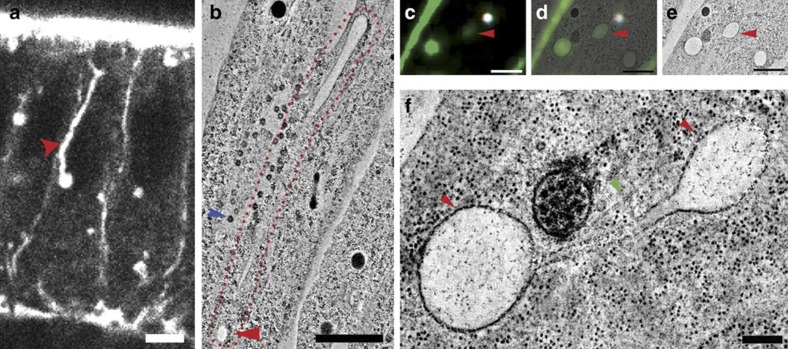
Rabankyrin-5 knock-down drives the elongation of apical endocytic tubes. (**a**) Rabankyrin-5 knock-down results in the elongation of endocytic tubules along the apico-basal axis. Single-plane two-photon optical cross-section of a Rabankyrin-5 siRNA embryo expressing sec::GFP showing a typical, elongated, sec::GFP-positive tubule (red arrow). Scale bar, 5 μm. (**b**) Ultrastructural characterization of an elongated endocytic tubule in a Rabankyrin-5 siRNA embryo. A single section from an EM tomogram of a Rabankyrin-5 siRNA embryo showing a typical elongated tubule that extends past the base of the nucleus (blue arrow indicates a nuclear pore at the base of the nucleus). Scale bar, 500 nm. (**c**–**f**) Ultrastructural characterization of elongated endocytic tubules in Rabankyrin-5 siRNA embryos. (**c**–**e**) Correlative light-EM of a basal sec::GFP structure in direct continuity with the elongated tubule shown in panel **b**. Fluorescence image of an EM section showing sec::GFP fluorescence (green) (**c**), a fluorescence/EM overlay (**d**) and a section from an EM tomogram of the same volume (**e**). Scale bar, 1,000 nm. (**f**) High-magnification tomographic section corresponding to panel **e** showing interconnected vesicular varicosities (red arrows that extend from the base of the elongated tubule shown in panel **b**. Vesicular varicosities of the elongated tubule exist in close proximity to microtubules (green arrow). Note, the sec::GFP-positive vesicular structure marked by the red arrows in **c**–**e** correspond to the basal vesicular varicosity marked by the red arrow in panel **b**. Scale bar, 200 nm.

The data presented here demonstrate that apical surface flattening is a dynamin-dependent morphogenetic process associated with an upregulation of tubular-endocytic intermediates and increasing levels of Rab5-positive endosomes. To identify proteins potentially involved in Rab5-positive endosome biogenesis, we carried out large-scale affinity chromatography and purified Rab5-specific effectors operating during these early stages of embryonic development. This experiment led to the identification of several proteins that were bound specifically to Rab5 in its active conformation (Fig. 5a). The most abundant effector identified by this approach was CG41099, the *Drosophila* homologue of human Rabankyrin-5 (Fig. 5a), a Rab5 effector linked to apical endocytosis in polarized MDCK cells^29^. In addition, Rabankyrin-5 has been shown to regulate macropinocytosis, a distinct form of endocytosis that involves the formation of large (0.2–10 μm) vesicular/vacuolar structures^29^. We therefore tested whether tubular endocytosis at the apical surface is regulated by Rabankyrin-5 through its interaction with Rab5. Simultaneous imaging of EGFP::Rabankyrin-5 and sec::mCherry revealed that Rabankyrin-5 associates with apical vacuolar structures positive for sec::mCherry (Fig. 5b–d). This result was confirmed using an antibody against *Drosophila* Rabankyrin-5 (Supplementary Fig. S4a). Moreover, correlative light-electron microscopy revealed that EGFP::Rabankyrin-5-positive membranes are organized as convoluted tubular structures (Fig. 5e–g). Live imaging of control embryos demonstrated that vacuolar structures formed at the apical plasma membrane and moved towards the basal side of the cell with long-range movements, which are presumably microtubule dependent. Conversely, in embryos expressing short hairpin RNAs against Rabankyrin-5 (the knock-down of Rabankyrin-5 was almost complete at both mRNA and protein levels, Supplementary Fig. S4b,c) this step was impeded resulting in the formation of long tubular membranes extending for over 15 μm along the apico-basal axis of the cell (Fig. 6a and Supplementary Fig. S4d–m and Supplementary Movies 8,9). Using correlative light-electron microscopy, we tracked one of these long tubular-endocytic membranes filled with endocytic cargo (Fig. 6b). This long tubule extended parallel to microtubules and its basal tip appeared composed of multiple varicosities interconnected by a constricted membrane domain (Fig. 6f and the tomogram depicted in Supplementary Movie 10). We speculate that, at the functional level, this terminal region may serve as a platform for the budding and generation of vacuoles, in a process equivalent to the formation of vacuoles from the shorter tubular invaginations seen in wild-type embryos (Fig. 2c–f). In support of this hypothesis, we observed the sequential budding of vacuoles from the tip of elongated tubes by live imaging in Rabankyrin-5-depleted embryos (Supplementary Movie 9). The elongation of tubular-endocytic membranes induced upon Rabankyrin-5 knock-down revealed that one role of the Rab5 machinery in this apical endocytic pathway is to control budding, and that other molecule/s must act upstream of Rabankyrin-5 in the initiation of tubular endocytosis and surface flattening.

### Dynamin is required for tubular membrane biogenesis

**Figure 7:**
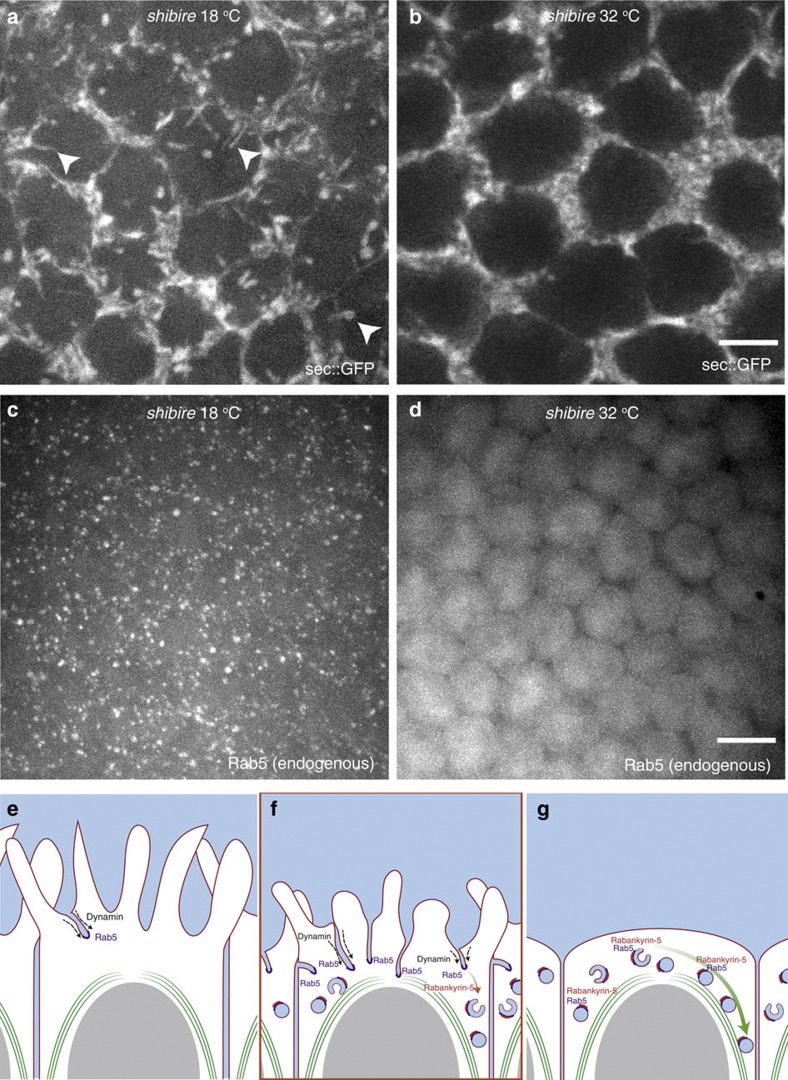
The biogenesis of apical endocytic tubules is regulated by dynamin. (**a**,**b**) Live imaging of internalized sec::GFP before cell flattening in shibire mutant embryos imaged at 18 °C (**a**) and at 32 °C (**b**), respectively. Multiple vacuolar and tubular structures were present in shibire mutant embryos imaged at 18 °C (white arrowheads). In contrast, shibire mutant embryos reared at the non-permissive temperature (32 °C) expressing sec::GFP showed no vacuolar or tubular structures. Scale bar, 5 μm. (**c**,**d**) Live imaging of GFP::Rab5 expressed at endogenous levels in shibire mutant embryos reared at the permissive temperature (18 °C, **c**) and at the non-permissive temperature (32 °C, **d**) during mid-cellularization. The punctate distribution of Rab5-positive endosomes was lost upon inhibition of dynamin activity and exhibited a diffuse cytoplasmic distribution. Scale bar, 5 μm. (**e**–**g**) A model for remodelling of the apical surface by tubular endocytosis. Early during cellularization (**e**) the apical surface is rich in villous protrusions. At this stage, the abundance of tubular structures and apical Rab5 endosomes is low. During mid-cellularization (**f**) the upregulation of Rab5-positive tubular-endocytic intermediates with a high surface to volume ratio allows for the rapid internalization of large amounts of membranes. These membranes are incorporated into Rab5-positive vacuoles that travel basally (green arrow, panel **g**), thus driving apical surface flattening (**g**). This tubular-endocytic pathway is dynamin dependent and controlled by the Rab5-effector Rabankyrin-5.

Given the effect of dynamin inhibition on apical surface flattening, we hypothesized that the high surface to volume ratio of tubular-endocytic intermediates provides an efficient means by which to rapidly internalize large amounts of membrane in order to facilitate plasma membrane remodelling during morphogenesis. If this hypothesis is correct, then blocking dynamin activity, which inhibits membrane flattening, should also inhibit tubular endocytosis. To test this hypothesis, we followed the internalization of sec::GFP in *shibire* mutant embryos. When embryos were heat-shocked before surface flattening, formation of sec::GFP-containing tubular structures was completely abolished (Fig. 7a,b) and protrusions failed to retract (Supplementary Fig. S5a,b). The above result shows that dynamin is required for tubular endocytosis. Consistently, expression of Dynamin::YFP in dextran-injected embryos revealed that dynamin localizes to the apical plasma membrane and at sites that tubular-endocytic membranes emerge from (Supplementary Fig. S6a–g). The activity of dynamin in this pathway is most likely distinct from its role in clathrin-coated vesicle formation as depletion of alpha-adaptin from early embryos did not interfere with either apical surface flattening or biogenesis of tubular-endocytic membranes (Supplementary Fig. S7a–d). Importantly and in agreement with the role of clathrin-coated vesicle-mediated endocytosis at the invaginating furrow during early cellularization^14^, alpha-adaptin-depleted embryos did not cellularize properly and the furrow was severely disorganized (Supplementary Fig. S7b).

Next, we asked whether dynamin is directly linked to the formation of Rab5 endosomes. We generated embryos expressing endogenously tagged GFP::Rab5 in a *shibire*^*ts*^ mutant background. Strikingly, shifting these embryos to the restrictive temperature during surface flattening resulted in the almost complete disappearance of Rab5-positive endosomes within 2–5 min (Fig. 7c,d). We conclude that dynamin is required for the initiation of tubular endocytosis and for the formation of Rab5 endosomes during surface flattening. The localization of Rab5 to tubular invaginations together with the rapid disappearance of Rab5 endosomes upon dynamin inhibition suggest that during surface flattening apical endosomes form directly at the plasma membrane rather than by fusion of incoming clathrin-coated vesicles.

## Discussion

In summary, our data demonstrate that apical surface flattening is a dynamin-dependent process associated with the dynamic regulation and functional re-organization of both endocytosis and endosome biogenesis. Dynamin activity is required for both the initiation of tubular endocytosis as well as for *de novo* formation of apical Rab5 endosomes. The elongation of tubular-endocytic membranes observed upon Rabankyrin-5 knock-down suggests that one role of the Rab5 machinery in this pathway is that of mediating the formation of endosomes from tubular invaginations. Taken together, these results are consistent with a model in which surface flattening is an endocytosis-dependent morphogenetic process driven by the rapid internalization of large quantities of plasma membrane via tubular-endocytic intermediates (see model in Fig. 7).

The results of our analysis show that dynamin is required for the biogenesis of tubular-endocytic structures at the apical plasma membrane as membrane tubulation is completely abolished in *shibire* mutant embryos. Although a clear reliance upon dynamin for clathrin-mediated endocytosis has been demonstrated previously^30^,^31^,^24^,^32^, its role in clathrin-independent endocytic pathways and in apical endocytosis is less clear. The requirement for dynamin in the tubular-endocytic pathway we have described here must, however, be distinct from its role in membrane fission. If dynamin were required for membrane fission, one would rather expect an elongation of tubular-endocytic intermediates upon blocking dynamin activity. We favour an alternative model that takes into account the role of dynamin in regulating actin dynamics^24^,^33^. Surface flattening involves a complete re-organization of the underlying actin cytoskeleton^8^,^17^ and actin dynamics are intimately linked to endocytosis^34^. Therefore, it is likely that the two processes are interconnected during apical surface flattening and coordinated by dynamin.

Elegant work in yeast has clearly demonstrated the requirement of actin during endocytosis, in particular for the invagination and scission of clathrin-coated vesicles^34^. In polarized MDCK cells, actin has been recently shown to be required for apical endocytosis^35^ under conditions that increase membrane tension^36^. Interestingly, during cellularization, flattening of the apical surface is concomitant with the establishment of apical adherens junctions and a progressive lowering in the concentration of actin^17^. The establishment of adherens junctions, which is expected to increase tension^1^, in combination with the lower levels of actin could, in principle, favour membrane tubulation by delaying budding. The elongation of the tubular-endocytic membranes that we observed in Rabankyrin-5-depleted embryos could interfere precisely with this mechanism.

Rabankyrin-5 has been previously shown to control macropinocytosis and apical pinocytosis in polarized MDCK cells^29^. Although the precise molecular mechanism underlying Rabankyrin-5 function is not known, its domain architecture and involvement in macropinocytosis suggests that it might coordinate the interaction between the actin cytoskeleton and membranes. Similarly, Rab5 was previously shown to act at the plasma membrane during clathrin-coated vesicle-mediated endocytosis and macropinocytosis^18^,^29^. Unfortunately, we could not directly test the function of Rab5 in promoting tubular endocytosis (Rab5 is maternally provided and is required during oogenesis). However, by generating a GFP-tagged Rab5 knock-in line we demonstrated a direct connection between Rab5 and endocytic tubules. More importantly, we demonstrated a precise correlation between the increase in apical Rab5 endosomes and restructuring of the apical surface. One possibility is that the upregulation of Rab5 might directly trigger the formation of tubular plasma membrane invaginations. Alternatively, Rab5 might be recruited to tubular invaginations only subsequent to their formation and control their post-internalization processing through Rabankyrin-5. This model is in accordance with both the elongation of endocytic tubules in Rabankyrin-RNAi embryos as well as with the loss of Rab5 endosomes and tubular invaginations caused by inhibiting dynamin activity (Fig. 7a–d).

Tubular endocytosis has been mostly characterized in mammalian cell culture where it has been associated with the internalization of GPI-anchored proteins, and mobilization of adhesion complexes^9^,^37^. This pathway is usually referred to as the CLIC (clathrin-independent carrier) pathway and is controlled by GRAF1^38^. The convoluted tubular morphology of the GFP-Rabankyrin-5 membranes (Fig. 5g) closely resembles that of previously described CLIC intermediates^39^. We do not know, however, whether these represent the same compartments, it is, however, likely that multiple tubular-endocytic pathways exist *in vivo*. For instance, during the remodelling of epithelial adherens junctions the internalization of E-cadherin has been shown to occur via tubular-endocytic intermediates in a pathway involving Dynamin, Cdc42 and its effector Cip4^2^,^40^,^41^. This tubular-endocytic pathway, however, seems distinct from the apical endocytic pathway described in this study, at least with respect to its regulation by Dynamin. Levayer *et al*.^2^ reported that blocking Dynamin activity during junction remodelling resulted in the elongation of tubular intermediates, thus suggesting that these membranes serve as budding sites for clathrin-coated vesicles. Our data instead demonstrate that upon shifting *shibire*^*ts*^ mutant embryos to the non-permissive temperature during apical surface flattening, membrane tubulation ceases immediately (Fig. 7). Importantly, we also show that tubular-endocytic membranes bud as a whole and lead to the formation of vacuolar Rab5-positive endosomes. This last result together with the identification of Rabankyrin-5 as a regulator of budding suggests that this pathway mechanistically resembles macropinocytosis. This is also in agreement with the requirement for Dynamin in the biogenesis of macropinsosomes^25^.

The activation of tubular endocytosis has been linked to the reduction of the apical surface area in cells undergoing apical constriction during morphogenesis^41^,^42^. Similarly, we also observed that the upregulation of tubular endocytosis is particularly prominent in ventral cells at the onset of ventral furrow formation. This morphogenetic movement is characterized by the synchronous invagination of ~1,000 cells, and is thought to be driven by the constriction of the apical surface of the internalizing cells^4^,^27^,^43^. The upregulation of apical endocytosis precedes any visible change in the reduction of the apical surface area. Therefore, it is possible that endocytosis acts upstream of apical constriction by controlling membrane uptake, thus facilitating the constriction of the actin–myosin network.

Besides controlling apical surface flattening, the internalization of apical membranes might also contribute to the lateral growth of the plasma membrane during cellularization. However, the tracking data presented in Fig. 3 suggest that it is unlikely that Rab5 endosomes contribute directly to lateral membrane growth as the majority of Rab5 particles move towards the basal side of the cell for only 15 μm without following the invagination of the furrow as one would have expected if Rab5 membranes contributed directly to membrane growth. It is more likely that Rab5 vacuoles mature into late endosomes and that the excess of apical membranes is degraded or stored in lysosomes. Previous data have suggested that apical membranes are continuously internalized during early cellularization and transported to the invaginating furrow via Rab11 recycling endosomes^15^. Our data do not directly address this model. Rather, our results reveal a novel function of apical endocytosis in regulating the morphology of the apical plasma membrane during epithelial morphogenesis. As a logical extension of this work, it will be interesting to test whether the mechanisms we have uncovered operate during other instances of epithelial remodelling during morphogenesis in other organisms, including vertebrates.

## Methods

### TIRF microscopy

Embryos were dechorionated with 20% sodium hypochlorite solution and positioned on glass-bottom culture dishes (MatTek) in a drop of PBS solution. In order to produce a uniform interface between the vitelline membrane and the coverslip, a thin slab (~5 mm) of 2% agar was placed on top of embryos before imaging. Time-lapse TIRF imaging was performed on an Olympus Biosystems Cell^R TIRF system using an Olympus APO N × 60 oil objective (NA 1.49). The incident angle was set at the critical angle for total internal reflection with subsequent small, manual angular adjustments used to optimize signal from the apical membrane. All imaging was performed at room temperature unless stated otherwise. For experiments involving the temperature-sensitive *shibire* mutant, either cold (18 °C) or pre-warmed (32 °C) PBS and agar were used for embryo mounting and live TIRF-M imaging was subsequently conducted in a temperature control chamber at either 18 or 32 °C.

### Confocal and 2-photon microscopy

Embryos were positioned on siliconized glass-bottom culture dishes (MatTek) and immersed in PBS solution. Imaging was performed with a spinning disk confocal Ultraview VOX system (Perkin Elmer) using a × 100 NA 1.3 oil immersion objective (Zeiss). Two-photon imaging was performed using Zeiss LSM 780 NLO system using a × 63 NA 1.2 water immersion objective (Zeiss).

### Correlative light-electron microscopy

Correlative light-electron microscopy of *Drosophila* embryos was performed as previously described for yeast^44^. Briefly, Rabankyrin::GFP-expressing embryos were first preserved at near-native conditions by high-pressure freezing using a high-pressure freezing machine (HPM 010; Bal-Tec). Embryos were subsequently processed by freeze substitution and embedding in Lowicryl HM20 (Electron Microscopy Sciences, Hatfield, PA) in an automated freeze substitution machine (AFS2; Leica). Freeze substitution was performed at −90 °C for 48–54 h with 0.1% (wt vol^−1^) uranyl acetate in glass-distilled acetone. The temperature was then raised to −45 °C at a rate of +5 °C per hour, and samples were washed with acetone and infiltrated with increasing concentrations (10, 25, 50 and 75%; 4 h each) of Lowicryl in acetone while the temperature was further raised to −25 °C. Lowicryl (100%) was exchanged three times in 10-h steps and samples were uiltraviolet polymerized at −25 °C for 48 h, after which the temperature was raised to 20 °C at a rate of +5 °C per hour and uiltraviolet polymerization continued for 48 h. Three hundred nanometre sections were cut with a microtome (Ultracut UCT; Leica) and a diamond knife (Diatome) and picked up on copper–palladium slot grids coated with separate layers of formvar and carbon. Blue (excitation 365 nm per emission 415 nm) 100 nm TetraSpecks (Invitrogen) were pretreated (to reduce fluorescence intensity) with 0.1% Tween-20 for 10 min, washed twice by ultracentrifugation at 100,000 *g*, resuspended in PBS and adsorbed to the EM grids by placing the grids section face-down onto a 15-μl drop of Tetraspecks for 10 min. The grids were then washed with three drops of water and blotted with filter paper. The embryo sections mounted on EM grids were placed on a droplet of water sandwiched between two glass coverslips and imaged face-down at room temperature using a widefield fluorescence microscope (model IX81; Olympus) fitted with a × 100, NA 1.45 objective, a camera (Orca-ER; Hamamatsu Photonics), and electronic shutters and filter wheels (Sutter Instrument Co). Fluorescence microscopy was accomplished with a lamp (X-Cite 120PC; EXFO Life Sciences) using 470/22, and 377/50-nm filters for excitation of GFP/TetraSpecks, and Tetraspecks alone, respectively. Emission was imaged using a 520/35-nm filter for GFP/TetraSpecks, and a 520/35-nm filter for TetraSpecks alone. The CCD camera, filter wheels and shutters were controlled by MetaMorph software (Universal Imaging Corp.).

### Electron tomography

Grids carrying embryo sections were post-stained with 2% uranyl acetate in 70% methanol and Reynolds lead citrate for contrast enhancement. Fifteen nanometre protein A–coupled gold beads were adsorbed on both sides of all grids as tomographic-fiducial markers. Grids were placed in a high-tilt holder (Model 2020; Fischione Instruments), and digital images were recorded on a camera (4 k Eagle; FEI) as dual-axis tilt series over a −60° to 60° tilt range (1° increment) on a microscope (Tecnai TF30; FEI) operated at 300 kV. Tomograms were reconstructed using the IMOD software package (version 3.13.2; Kremer *et al*., 1996)^45^. Fiducial-based correlation was performed exactly as previously described^44^.

### Fly stocks

WT flies were Oregon-R; all stocks were maintained by standard methods at 25 °C, unless otherwise specified. y,w*;P[UASp>YFP::Rab5] (BL-9775). y,w*; EGFP::Rab5/CyO (endogenous). y,w*;;P[UASp>sec::mCherry]. y,w*,P[UASp>sec::GFP/Y]. y,w*;;P[UASp>sec::GFP]. y,sc*,v1; P[TRiP.HMS01228]attP2/TM3, Sb (CG41099 siRNA BL-34883). y,sc*,v^1^; P[YFP-RNAi]attP2 (TRIP). y,w*;P[UASp>EGFP::CG41099-PC]. w*; P[mat α Tub>Gal4::VP16];[mat α Tub>Gal4::VP16]. w*;; P[Sqh>Gap43::mCherry]. Shibire^TS^ (BL-7068). y,w*;P[UASp>Dynamin::YFP]. w^1118^; P[UAS>EB1::GFP].

### GFP::Rab5 homologous recombination

To generate GFP::Rab5 expressed at endogenous levels we adapted the ends-out homologous recombination method^28^. An pEOC (pEndsOutmCherry) targeting vector was designed on the basis of the pRK1 vector by replacing the negative selection marker UAS-Rpr with 3xP3::mCherry^46^ for more efficient screening purposes. In addition, the multiple cloning site was replaced by the *Not*I/*Mlu*I/*Eco*RI/*Sph*I/*Bgl*II/*Bsi*WI and the *Aat*II/*Nde*I/*Nae*I sites. Homologous recombination arms (5′ 3.2 kbp and 3′ 2 kbp) were amplified from BACMID DNA containing the Rab5 locus (BACR22P10, BPRC). In addition, EGFP with a terminal glycine–alanine–glycine–alanine linker was inserted at the 5′-end of the Rab5-coding sequence. Homologous arms were cloned into the pEOC vector and a homologous recombination targeting line on the third chromosome was generated. This targeting line was subsequently crossed to the 6939-hid line and the crossing scheme was followed as described by Huang *et al*.^28^. Recombinant flies with the white marker segregating to the second chromosome were selected. The selection of false-positive insertions was performed by a screen against mCherry expression in the 3xP3 promoter pattern^47^. The white marker was removed using the Cre/loxP system. Correct insertion was confirmed by sequencing.

### Cloning and antibody generation

UAS-secGFP and UAS-secmCherry were generated by fusion of the coding sequence of the folded gastrulation signal peptide (amino acids 1–22) to the fluorescent proteins EGFP and mCherry, respectively, and cloned into the pPW vector (DGRC) using the Gateway cloning system (Life Technologies). cDNA for CG41099-PC (D-Rabankyrin) was reverse-transcribed from total RNA extracted from 0–4 h-old embryos. D-Rabankyrin was cloned into the pPGW vector (DGRC) using the Gateway cloning system (Life Technologies). Antibodies against CG41099 were made in rabbit as follows: A fragment corresponding to amino acids 660–920 of CG41099-PC was cloned into the pET32 vector (Novagen) using *Bam*HI and *Xho*I restriction sites. Protein was purified from BL21 E.coli cells (Stratagene) and sent for antibody production (Eurogentec). Antibodies were subsequently purified using affinity chromatography.

### Western blot and immunohistochemistry

For detection of endogenous Rabankyrin-5 protein 10 cellularizing embryos were lysed in SDS lysis buffer. Western blotting was achieved using the western lightning ECL kit (Perkin Elmer) according to the manufacturer’s protocol. Rabbit anti-CG41099 (1:5,000) and mouse anti-alpha-Tubulin (1:10,000, Sigma-Aldrich, clone B-5-1-2) antibodies were used. Embryos were dechorionated for 3 min in 20% sodium hypochlorite solution and fixed in 4% paraformaldehyde (Electron Microscopy Sciences) and heptane (Sigma) for 20 min. Fixed embryos were incubated with an anti-CG41099 antibody (1:250). Alexa 488 (company) anti rabbit secondary antibodies were used (1:500). Imaging was performed with a spinning disk confocal Ultraview VOX system.

### Quantitative PCR

RNA was extracted from 10 cellularizing embryos using the RNeasy Mini Kit (Qiagen) with additional DNA digestion performed ‘on-column’ using RNase-Free DNase (Qiagen). cDNA was synthesized with the Superscript III First strand synthesis System (Life Technologies). qRT–PCR for CG41099 was performed with the SYBR Green PCR master mix (Applied Biosystems) using an Applied Biosystems 7500 Real-Time PCR System with the following primers: (CG41099_1) 5′-CAGGGTGCAGACATTACAGC-3′ (CG41099_2) 5′-CGGACCATAACGGTGATTCT-3′ and was normalized to the RPL32 gene with following primers: (RPL32_1) 5′-GCTAAGCTGTCGCACAAA-3′ (RPL32_2) 5′-TCCGGTGGGCAGCATGTG-3′. qRT–PCR for α-adaptin was performed with the SYBR Green PCR master mix (Applied Biosystems) using standard protocols with the following primers: α-adaptin_1 5′-GTCTGGATTGGGATCCACTT-3′ α-adaptin_2 5′-CTTAGCGGTGAACAACAACG-3′ and was normalized to the *RPL32* gene with following primers: RPL32_1 5′-GCTAAGCTGTCGCACAAA-3′ RPL32_2 TCCGGTGGGCAGCATGTG.

### Quantification and statistics

Quantification of intracellular signal in UAS:secGFP/+; Tub67::GAL4/+; Tub67::GAL4/+ embryos was performed manually. Intracellular tubes and vesicles positive for sec::GFP were counted from the Z-stack corresponding to the volume between 4–5 μm under the apical membrane over the course of cellularization. Quantification was performed on three areas of 25 × 25 μm^2^ each (625 μm^2^) in three different embryos.

### GFP::Rab5 Signal quantification

GFP::RAB5 signal at the apical plasma membrane was imaged over the course of cellularization using TIRF microscopy and subsequently quantified using CellProfiler^48^. Briefly, three independent data sets were obtained each for both ectopically expressed YFP::RAB5 and for endogenous GFP::RAB5. Identification and segmentation of RAB5-positive puncta was accomplished using cell profiler, registration was verified manually, and the number and integrated intensity of puncta was quantified. Both the number and integrated intensity of puncta were normalized by expressing their value over cellularization as a ratio to the mean value of the first 20 frames in each sequence.

### Rab5 particle tracking

A single-plane, sagittal cross-section time-lapse series was acquired during cellularization in a YFP::Rab5-expressing embryo by confocal microscopy (Perkin Elmer, Ultraview VOX system). YFP::Rab5 puncta were first segmented in cell profiler^48^ using the Robust Background Global thresholding method in Identify Primary Objects module. Segmented YFP::Rab5 particles were subsequently tracked in Imaris. Briefly, YFP::Rab5 particle tracks were filtered based on the criteria of having a duration of six frames or more, and having an origin within 5 μm of the apical plasma membrane. Resulting tracks were divided into tracks that persist within 5 μm of the apical surface, and those that descend basally past 5 μm of the apical plasma membrane.

### Quantification of the rate of apical surface flattening

First the images where corrected for bleaching by fixing the mean intensity at each time point to that of the initial image. Then the images where filtered using a mexican hat filter with values sigmax=7, sigmay=1, which was rotated according to angles in an interval from 0 to pi. The response images form a stack, where each plane corresponds to an angle from the interval, enhancing protrusions along the given angle. The maximum intensity projection of that stack was then segmented using Ilastik^49^ to yield a segmentation of the image. The skeleton of the image was then taken to compute the area in the image covered by protrusions.

### Purification of Rab5 effectors from cellularizing *Drosophila* embryos

Rab5 effectors were purified as previously described by Christoforidis and Zerial^50^ with the following modifications. Briefly, 0–4 h embryos were harvested, dechorionated for 2 min in bleach, washed in PBS 0.1% Triton X-100 and frozen at −80 °C. Thirty grams of packed embryos were diluted in 60 ml of lysis buffer and homogenized in a Dounce tissue grinder at 4 °C and processed as described by Christoforidis and Zerial^50^. Thereafter, the cytosolic fraction was divided in two aliquots and each aliquot was incubated with 1 ml of packed GST-Rab5 beads loaded with either GDP (inactive) or GTP-γS (active). All the subsequent steps were performed as described by Christoforidis and Zerial^50^.

### Dextran injections

In all, 10,000 MW 647 Dextran, and pHrodo Red (Life Technologies) were injected into the perivitelline space of cellularizing embryos as described previously by Levayer *et al*.^2^ Embryos were covered with a thin layer of halocarbon oil 700/27 (1:2) (Sigma). The coverslip was placed on a microscope slide platform and embryos were visualized using a standard 18 upright microscope equipped with a × 10 objective (Zeiss). Microinjection was carried out with an Eppendorf 5242 microinjector. Microinjection pipettes were pulled from borosilicate glass capillaries (1.2 mm outer diamater × 0.94 mm inner diamater, Harvard Apparatus), using a P-97 Flamming/brown puller (Sutter Instrument Co). Here 20 mg ml^−1^ of dextran in PBS solution was used.

## Author contributions

The experiments were conceived and designed by P.F., A.S.N. and S.D.R. P.F. developed the TIRF-M imaging protocol, generated the endogenously tagged GFP::Rab5 flies and performed the live imaging analysis together with A.S.N. and S.M. A.S.N. developed the correlative-light microscopy method in the early *Drosophila* embryo and performed the electron microscopy and tomography analysis with guidance from J.A.G.B. E.L. preformed the biochemical purification of the Rab5 effectors and A.R. performed the dextran injection experiments and two-photon movies. S.S. analysed the data shown in fig. 1e–h and generated the graph shown in fig. 1i. P.F., A.S.N. and S.D.R. analysed together all the data and wrote the manuscript together with J.A.G.B.

## Additional information

**How to cite this article:** Fabrowski, P. *et al*. Tubular endocytosis drives remodelling of the apical surface during epithelial morphogenesis in *Drosophila*. *Nat. Commun.* 4:2244 doi: 10.1038/ncomms3244 (2013).

## Supplementary Material

Supplementary Figures Supplementary Figures S1-S7

Supplementary Movie 1 Remodeling of the apical plasma membrane over the course of cellularization imaged by TIRF-M. GAP43::mCherry (white). Scale bar, 5 μm.

Supplementary Movie 2 Microtubule dynamics inside of apical protrusions during cellularization imaged by TIRF-M. EB1::GFP (green), GAP43::mCherry (purple). Scale bar, 5 μm.

Supplementary Movie 3 Blockage of apical surface flattening in a shibire mutant embryo over the course of cellularization imaged by TIRF-M at 32 °C. GAP43::mCherry (white). Scale bar, 5 μm.

Supplementary Movie 4 Endocytic tubule dynamics over the course of cellularization imaged as a single-plane, subapical section by spinning disc confocal microscopy. sec::GFP (white). Scale bar, 5 μm.

Supplementary Movie 5 Dynamics of endogenous Rab5 at the apical plasma membrane over the course of cellularization imaged by TIRF-M. GFP::RAB5 (white). Scale bar, 5 μm.

Supplementary Movie 6 Dynamics of endogenous Rab5 imaged as a saggital cross section over the course of cellularization imaged by 2-photon microscopy. GFP::RAB5 (white). Scale bar, 5 μm.

Supplementary Movie 7 Dynamics of Rab5 during mid-cellularization imaged as a sagittal embryo cross-section by spinning disc confocal microscopy. YFP::Rab5 (white). Scale bar, 5 μm.

Supplementary Movie 8 Endocytic tubule dynamics in a wild type embryo over the course of cellularization shown as a 5 μm z-projection of a sagittal embryo section imaged by two-photon microscopy. sec::GFP (white). Scale bar, 5 μm.

Supplementary Movie 9 Endocytic tubule dynamics in a Rabankyrin-RNAi embryo over the course of cellularization shown as a 5 μm z-projection of a sagittal embryo section imaged by two-photon microscopy. sec::GFP (white). Scale bar, 5 μm.

Supplementary Movie 10 EM Tomogram of an elongated endocytic tubule in a Rabankyrin-RNAi embryo (black arrows). Note the varicosity at the base of the tubule (red arrow). Scale bar, 500 nm.
