# Supplementary material for: Tubular endocytosis drives remodelling of the apical surface during epithelial morphogenesis in Drosophila
Source: Nat Commun. 2013 Aug 7;4:2244. doi: 10.1038/ncomms3244 (PMC3753550; doi:10.1038/ncomms3244)
Supplement: Supplementary Figures — S1-S7 [file ncomms3244-s1.pdf]

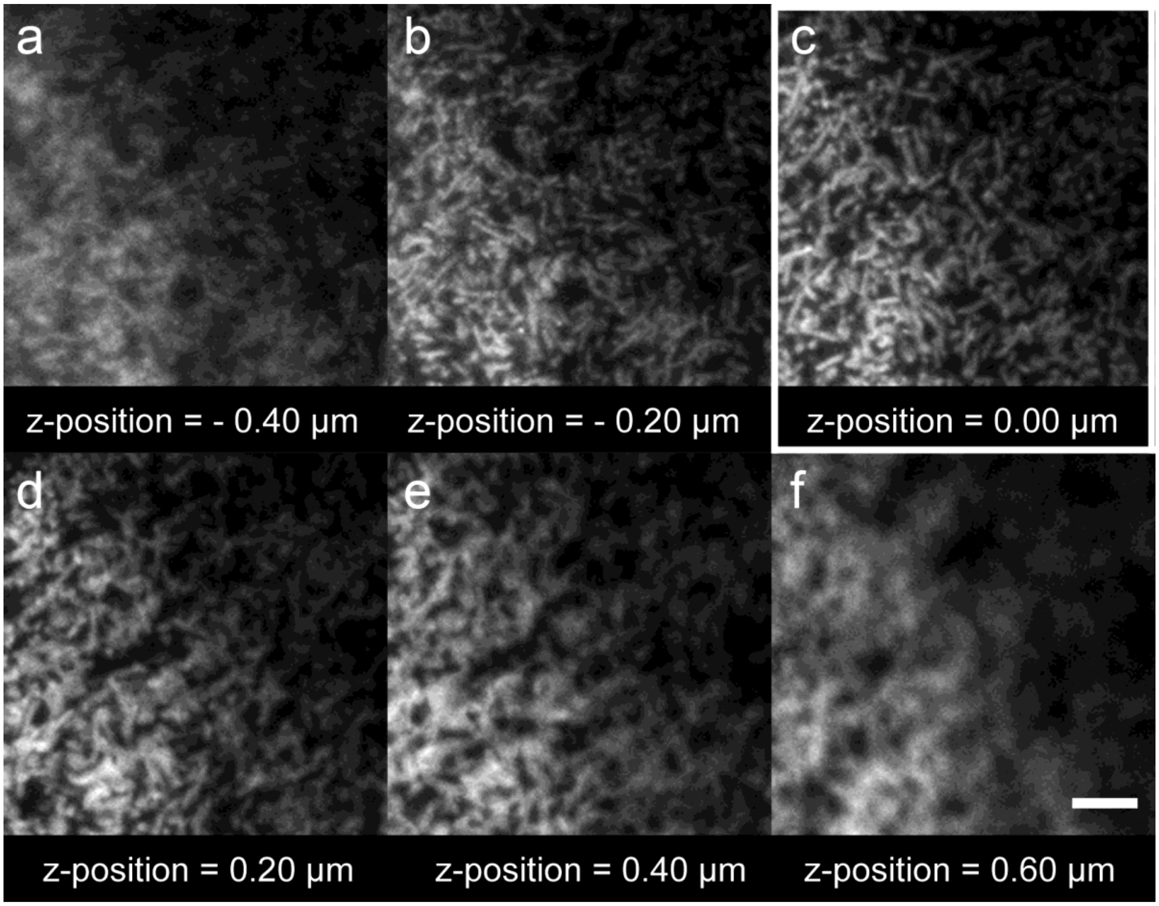

Supplementary Figure S1. In TIRF Microscopy only a thin, 0.4 μm plane is in focus.

TIRF imaging of the apical plasma membrane of a GAP43::mCherry-expressing cycle 14 Drosophila embryo at incremental focal depths is shown (panels **a-f**). Frames correspond to focal depths of - 0.4 (**a**), - 0.2 (**b**), 0.0 (**c**, white box), 0.2 (**d**), 0.4 (**e**), and 0.6 (**f**) μm, respectively. Note that images start to become blurred at a depth of +/- 0.2 μm from the focal plane of the super critical angle required to generate the evanescent wave. Scale bar, 5 μm.

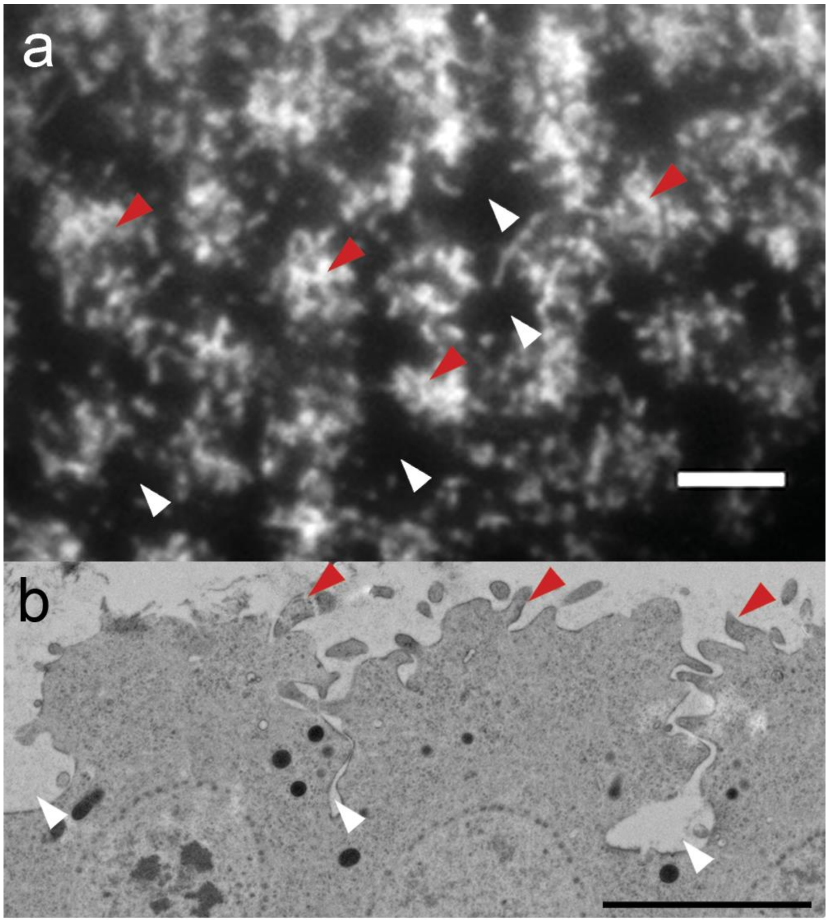

Supplementary Figure S2. Dark regions in TIRF imaging correspond to extracellular spaces.

(**a**) Imaging of villous protrusions at the apical plasma membrane during early cellularization with TIRF microscopy. Apical villous protrusions (red arrows), marked with GAP43::mCherry, are shown in white, whereas the extracellular spaces appear as dark regions (white arrows). Scale bar 5 μm. (**b**) Electron micrograph of a cross-section of an embryo at early cellularization showing furrow position (white arrows) with respect to apical villous protrusions (red arrows). Furrow depth is approximately 8 μm from the apical surface. Note that panels A and B correspond to the same stage but not to the same embryo. Scale bar 5 μm.

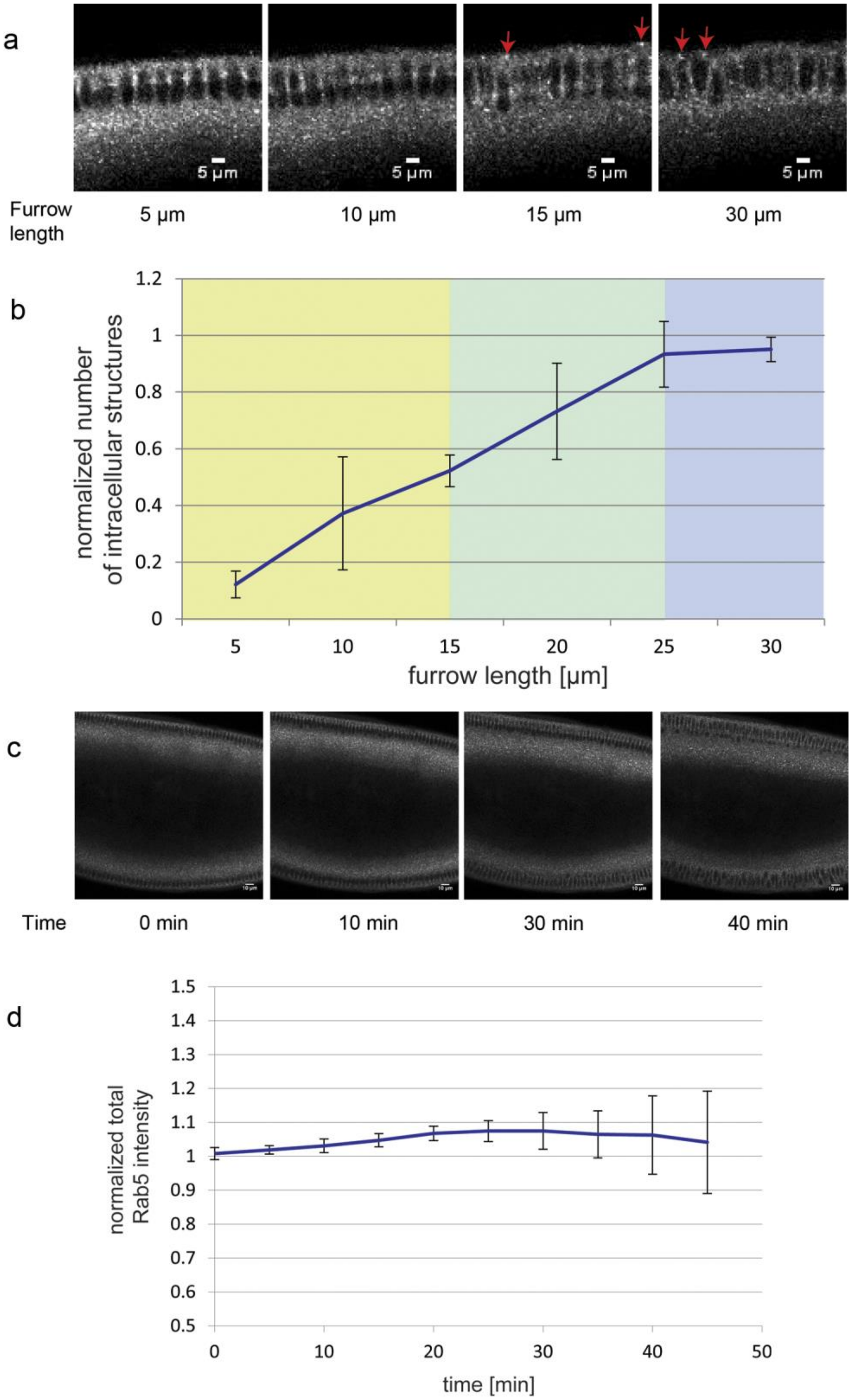

Supplementary Figure S3. Apical Rab5 endocytosis is upregulated during cellularization

(**a-b**) Quantification of endogenously tagged Rab5 endosomes originating from the apical plasma membrane over the course of cellularization. (**a**) 2-photon cross-section snapshots corresponding to 4 progressive stages of a cellularizing GFP::Rab5 expressing embryo. Embryo stage, denoted by furrow position from the apical surface, is shown at bottom. Apical Rab5 endosomes (red arrow) appear during mid-late cellularization (furrow length > 15 µm). Scale bar, 5 µm. (**b**) The early (yellow), mid- (green), and late (purple) stages of cellularization are highlighted. The number of Rab5 endosomes present within 4 micrometers from the apical plasma membrane was quantified from a single plane in three independent embryos using two photon microscopy (see Supplementary Movie 6). Standard deviation is shown as black lines. p-value = 0.001 (ANOVA).

(**c-d**) Total Rab5 signal does not change over the course of cellularization. (**c**) 2-photon cross-section snapshots corresponding to 4 progressive stages of a cellularizing GFP::Rab5 expressing embryo imaged at low time resolution (5 min) to minimize photobleaching. Embryo stage, represented as minutes from the onset of cellularization, is shown at bottom. Scale bar, 10 µm. Note, these imaging conditions, in which it was possible to visualize the entire embryo, do not provide adequate resolution with which to discriminate individual apical endosomes. (**d**) Quantification of total GFP::Rab5 intensity over the course of cellularization. The total GFP::Rab5 signal was quantified from a single plane using 2-photon microscopy in 5 independent embryos. Intensity values were calculated by quantifying the total fluorescence intensity of the apical 30 µm of the embryo at 5 minute intervals over the course of cellularization. Values shown represent the total fluorescence intensity at each time point normalized to the value of the first image acquired at the beginning of cycle 14. Standard deviation is shown as black bars. The change in signal intensity over time is not statistically significant (p-value = 0.74 ANOVA).

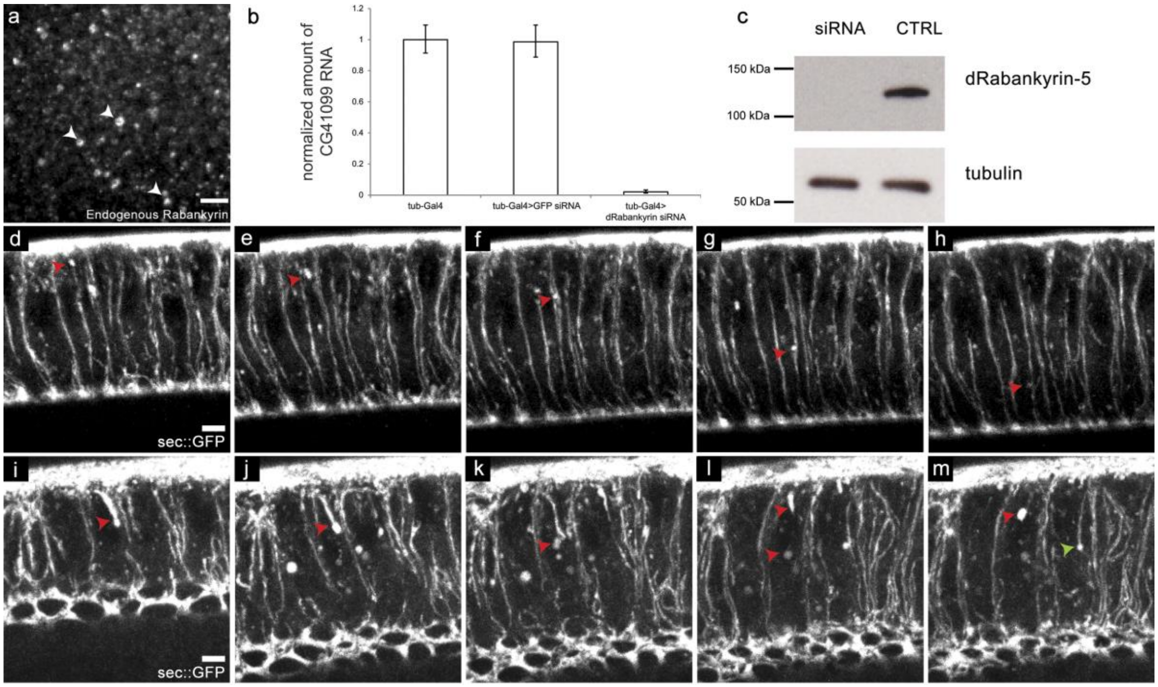

Supplementary Figure S4. Rabankyrin-5 knock-down drives the elongation of apical endocytic tubes

(**a**) Immunohistochemistry performed against endogenous Rabankyrin-5 shows that Rabankyrin-5 localizes to apical vacuolar structures (white arrows).
Scale bar, 5 μm
(**b-c**) Expression of Rabankyrin-5 siRNA results in specific and effective knock-down of endogenous Rabankyrin-5 mRNA and protein levels. (**b**) qRT-PCR analysis of Rabankyrin-5 siRNA-expressing embryos showed a 98% reduction of the level of Rabankyrin-5 mRNA in comparison to either a GFP siRNA-expressing line or to the GAL4 driver line alone. N=3 for each genotype. Standard deviation is shown as black bars. (**c**) Western-blot analysis of Rabankyrin-5 endogenous protein levels in Gal4 driver and in Rabankyrin-5 knock-down lines. Rabankyrin-5 protein is undetectable in cellularizing embryos expressing siRNA targeting the gene (lane 1; siRNA) compared to GAL4 driver line embryos (lane 2; CTRL). Lower panel shows alpha-tubulin signal as a loading control.
(**d-m**) Two-photon optical cross section of wild type sec::GFP embryos (**d-h**) and Rabankyrin-RNAi; sec::GFP embryos (**i-m**) showing the dynamics of endocytic tubule formation. In wild type sec::GFP embryos budding of sec::GFP-positive vesicles from the apical surface was frequently observed whereas endocytic tubules were never observed to extend past the base of the nuclei. Red arrowheads mark vacuoles filled with sec::GFP moving towards the base of the cells. Timepoints correspond to t=0s (**d**), t=110s (**e**), t=160s (**f**), t=195s (**g**), t=235s (**h**). In contrast, in Rabankyrin-5-RNAi embryos, sec::GFP-positive endocytic tubes that extend past the base of the nuclei were frequently observed (k, red arrow; m, green arrow). Red arrowheads indicate maturation of the elongated intracellular tubes marked with sec::GFP. Timepoints correspond to t=0s (**i**), t=225s (**j**) , t=300s (**k**) , t=525s (**l**), t=550s (**m**).

Scale bar, 5 µm.

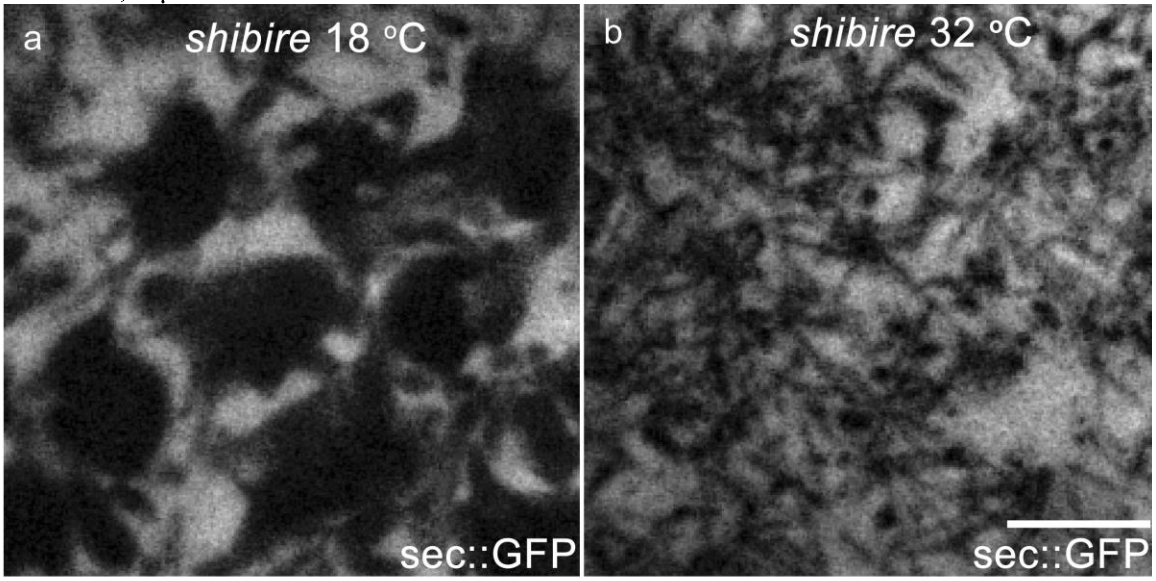

Supplementary Figure S5. Visualization of apical protrusions in a *shi*^{ts} mutant background using confocal microscopy

(**a-b**) Live imaging of the apical plasma membrane counterstained with soluble sec::GFP in shibire mutant embryos imaged at 18 °C (**a**) and 32 °C (**b**). The extracellular space is filled with sec::GFP and is shown in white. The apical cell surface is shown in black. Flattening of the apical plasma membrane was observed at the permissive temperature (**a**) whereas at the non-permissive temperature surface flattening was impeded. White arrows point to the villous protrusions that did not retract. 1 um z-projection of the apical surface is shown. Scale bar, 5 µm.

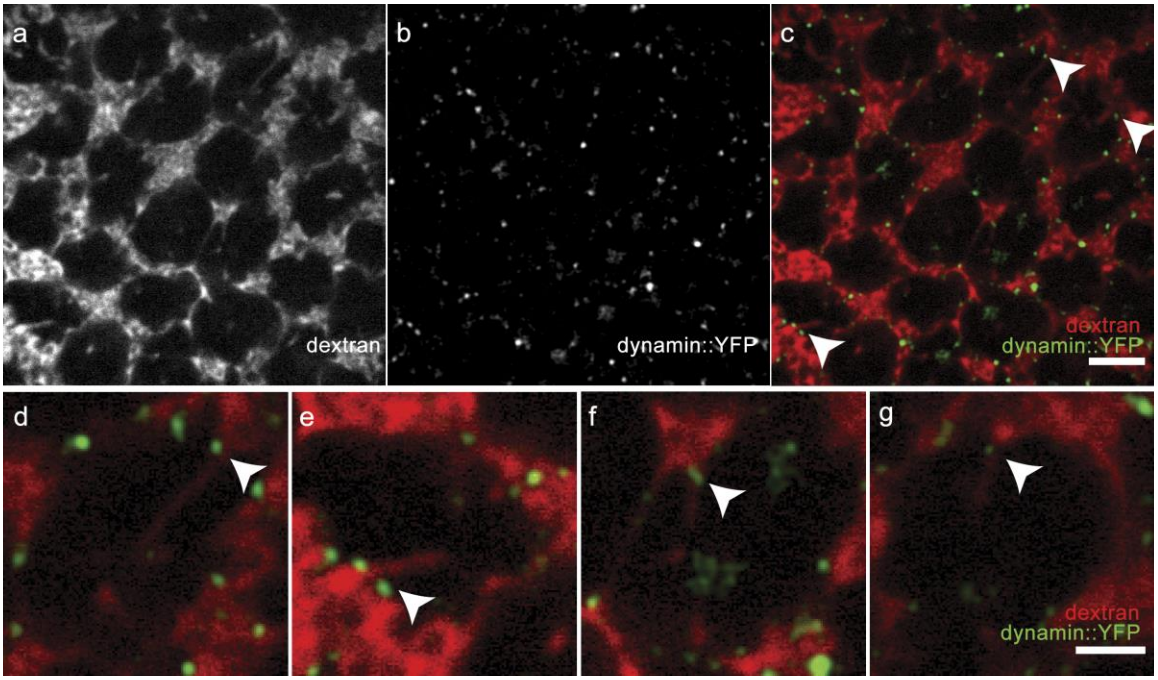

Supplementary Figure S6. Dynamin localizes to endocytic tubes

(**a-g**) Ectopic expression of dynamin::YFP injected with fluorescently labeled dextran shows co-localization of dynamin with endocytic tubes originating from the plasma membrane (white arrows). Single plane images of soluble cargo (fluorescently labeled dextran) (**a**) and dynamin::YFP (**b**). Overlay of fluorescently labeled dextran (red) with dynamin:YFP (green) showing co-localization at the neck of the endocytic tubes (**c**). (**a,c**) Scale bar, 5 µm
(**d-g**) High magnification view of endocytic tubes decorated with dynamin::YFP from panel c. Dynamin::YFP (green) localizes to the neck of the endocytic tubes (white arrows) originating from the apical plasma membrane filled with fluorescently labeled dextran (red). (**d-g**) Scale bar, 2 µm

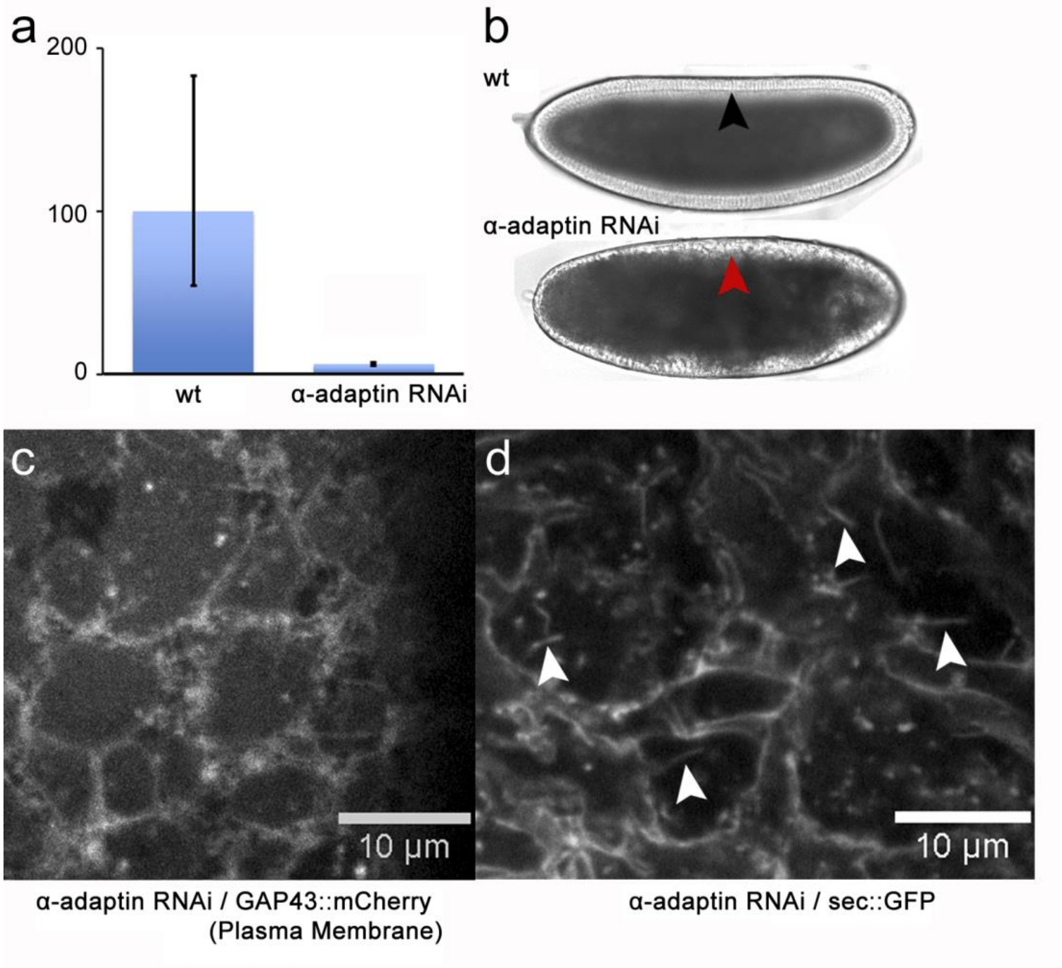

Supplementary Figure S7. α-adaptin function is not required for surface flattening or the biogenesis of endocytic tubes

(**a**) α-adaptin shRNA mediates effective knock-down of α-adaptin RNA. Quantification by RT-PCR in control (67.15) and α-adaptin shRNA (67.15; UAS-α-adaptin shRNA) embryos reveals a 93% knockdown in α-adaptin abundance. n = 3 embryos for each genotype. Standard deviation is shown as black bars. (**b**) α-adaptin is required for cellularization. Whereas wildtype embryos (wt) show uniform furrow ingression (black arrow), embryos expressing α-adaptin shRNA display a penetrant defect in cellularization with stereotypical irregularities in furrow ingression (red arrow). (**c**) α-adaptin is not required for surface flattening.The apical plasma membrane flattens during cellularization in α-adaptin shRNA embryos. Membranes are labeled with GAP43::mCherry and the apical plasma membrane was imaged by TIRF-M. Scale bar, 10 µm.
(**d**) α-adaptin is not required for the biogenesis of endocytic tubes. Endocytic tube biogenesis is observed during cellularization in embryos expressing α-adaptin shRNA. Sec::GFP is shown in white. A single plane obtained by confocal microscopy is shown. Scale bar, 10 µm.
